# Downregulation of CYP2E1 is associated with poor prognosis and tumor progression of gliomas

**DOI:** 10.1002/cam4.4320

**Published:** 2021-10-06

**Authors:** Liguo Ye, Yang Xu, Long Wang, Chunyu Zhang, Ping Hu, Shi’ao Tong, Zhennan Liu, Daofeng Tian

**Affiliations:** ^1^ Department of Neurosurgery Renmin Hospital of Wuhan University Wuhan Hubei P.R. China

**Keywords:** biomarker, CYP2E1, ferroptosis, gliomas, lipid metabolism

## Abstract

**Objective:**

To explore the role and possible regulatory mechanisms of CYP2E1 in gliomas.

**Methods:**

RNA‑seq data and corresponding clinical information of glioma patients were collected from The Cancer Genome Atlas and Chinese Glioma Genome Atlas, and mRNA data of normal brain tissues were obtained by the Genotype‐Tissue Expression project. The Wilcoxon test was performed to analyze the correlation between CYP2E1 expression and glioma subtypes. Univariate and multivariate Cox proportional hazards regression, receiver operating characteristic curves, and Kaplan–Meier plots were used to evaluate the prognostic value of CYP2E1 in glioma. Functional enrichment analyses and immune infiltration analyses were performed to investigate the potential function of CYP2E1 in gliomas. Moreover, we investigated the miRNA and epigenetic mechanisms that regulate CYP2E1 expression. Finally, network pharmacology and molecular docking experiments were used to predict drugs that target CYP2E1.

**Results:**

The downregulation of CYP2E1 expression may predict a poor prognosis for glioma patients. CYP2E1 expression decreased with increasing WHO grade (II–IV), and its level was correlated with clinical features, including age, 1p19q codeletion status, and IDH state in glioma tissues. Furthermore, CYP2E1 was involved in lipid metabolism and ferroptosis and related to the tumor immune microenvironment due to its strong correlation with the levels of infiltrating monocytes and Tregs. Moreover, variation in the total methylation level and copy number of CYP2E1 was moderately correlated with its mRNA expression (*p* < 0.05). CYP2E1 was predicted to be targeted by hsa‐miR‐527, whose expression was negatively related to CYP2E1 mRNA expression (*p *< 0.05). In addition, effective compounds that target CYP2E1, including 18beta‐glycyrrhetinic acid, styrene, toluene, nicotine, m‐xylene, p‐xylene, and colchicine, were identified.

**Conclusion:**

The downregulation of CYP2E1, which affects lipid metabolism and the ferroptosis signaling pathway, promotes the progression of gliomas.

## Graphical abstract

First, we identified that the downregulation of cytochrome P450 family 2 subfamily E member 1 (CYP2E1) in gliomas, CYP2E1’s expression level decreased with the degree of malignancy of gliomas. Then, based on functional enrichment analysis of CYP2E1 in TCGA‐glioma, the association between CYP2E1 and tumor immune microenvironment, lipid metabolism, and ferroptosis were explored to explain these findings could translate to a novel strategy to treat glioma patient with gliomas. Moreover, the total methylation level and copy number variation of CYP2E1 were moderately correlated with its mRNA expression (*p* < 0.05). CYP2E1 was predictively targeted by has‐miR‐527, which was negatively related to CYP2E1 mRNA expression (*p *< 0.05), which could explain the possible regulatory mechanism of CYP2E1 down‐regulation in gliomas. In addition, through network pharmacology and molecular docking, we found seven ingredients of Chinese traditional medicine targeting CYP2E1. In conclusion, this research identified a novel lipid metabolism and ferroptosis‐related marker in glioma. It's the first research to explore the role of CYP2E1 in gliomas.

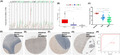

## 1 INTRODUCTION

Glioma is a malignant primary brain tumor originating from astrocytes or oligodendrocytes, representing 80% of all malignant brain tumors.^1^ The 5‐year survival rate of patients with glioma is <10%, and the most aggressive glioblastoma (GBM) has a 5‐year survival rate of <5%.^2^ Although significant progress has been made in deciphering the molecular pathogenesis of glioma in the past decade, it remains insufficiently understood due to the complexity of glioma.^3^, ^4^ By regulating multiple biologic processes, such as invasion, angiogenesis, immunosuppression, chemoresistance, and glioma stem cell phenotype induction, the tumor microenvironment (TME) has been shown to be related to tumor malignancy.^5^ Metabolic reprogramming, such as changes in lipid metabolism, occurs in the TME and impacts the proliferation, invasion, and metastasis of cancers.^6^, ^7^ In addition, changes in lipid metabolism are implicated in the modulation of tumor‐infiltrating immune cell (TIIC) antitumor immunity.^8^, ^9^, ^10^ Hence, discovering vital lipid metabolism‐related biomarkers and examining the association between markers and the tumor immune microenvironment (TIME) could facilitate the diagnosis and treatment of gliomas.

Cytochrome P450 family two subfamily E member 1 (CYP2E1), which plays an essential role in metabolizing alcohol,^11^, ^12^ can produce acetaldehyde and highly reactive oxygen species, which induce oxidative stress and increase lipid level.^13^ In addition, CYP2E1 is reported to generate reactive oxygen species (ROS) and nitric oxide through the induction of NADPH/xanthine oxidase and nitric oxide synthase in normal neurons.^14^ It has been reported that ferroptosis is a form of iron‐dependent oxidative cell death mediated by ROS accumulation and lipid peroxidation.^15^ When ROS levels continue to rise beyond the tolerance threshold of tumor cells, ferroptosis is triggered.^16^ In addition, ROS are highly associated with the immune response, cellular damage, and inflammatory disease.^17^ Numerous studies have shown that CYP2E1 plays a vital role in the occurrence and development of some solid tumors, such as liver cancer and childhood rhabdomyosarcoma,^18^, ^19^ and has some influence on the metabolism of antitumor drugs.^20^ However, the roles of CYP2E1 as a tumor suppressor or oncogene in glioma are still elusive, and its relevant regulatory mechanism and complex regulatory network still need to be fully elucidated.

In this study, related systematic research was conducted on the role of CYP2E1 in glioma. First, the characteristics of glioma samples’ clinical and molecular subtypes could be well stratified by CYP2E1 expression. Furthermore, through TIME analysis, the association between CYP2E1 and the infiltration level and abundance of TICs was investigated. Finally, the potential function of CYP2E1 in signaling pathways, such as those related to ferroptosis and lipid metabolism, was investigated through single sample gene set enrichment analysis (ssGSEA). In summary, the results may provide novel insight into glioma malignancy and immunotherapy.

## 2 MATERIALS AND METHODS

### 2.1 Patient samples

The Institutional Ethics Committee approved this study of the Faculty of Medicine at Renmin Hospital of Wuhan University. Informed consent was obtained from all the patients whose tissues were used. In total, six control samples from patients with cerebral hemorrhage, 24 samples from patients with low‐grade glioma (World Health Organization [WHO] grade II–III), and 40 samples from patients with GBMs were collected during May 2019 and April 2021. No patients were treated with chemotherapy or radiotherapy before surgery.

### 2.2 Publicly available database

RNA‑seq data and corresponding clinical information of glioma patients were collected from The Cancer Genome Atlas (TCGA) (http://cancergenome.nih.gov/), and the mRNA‐seq data of normal brain tissues were obtained from the Genotype‐Tissue Expression (GTEx) project. Then the mRNA data of TCGA and GTEx were merged and normalized by R package “limma.” Similarly, the RNA‐seq and clinical information obtained from the mRNAseq_693 and mRNAseq_325 data sets in the Chinese Glioma Genome Atlas (CGGA) (http://www.cgga.org.cn) were merged and normalized as a validation set. Here, we used the “normalizeBetweenArrays” function of R package limma to remove multiple batch effects among different data sets.^21^, ^22^ All samples from patients aged <18 years, survival time shorter than 3 months, and incomplete information were removed. The training set included a total of 587 glioma tissues (including WHO grade II–IV) and 1152 normal brain tissues, and the validation set included a total of 681 samples.

### 2.3 The expression of CYP2E1 in tumor and normal tissues

The expression levels of CYP2E1 mRNA in pan‐cancer were investigated in the GEPIA database (http://gepia.cancer‐pku.cn/detail.zphp), and the values of CYP2E1 in different grade gliomas and normal brain tissues were compared in the training set and validation set by the R package limma;^22^ a *p*‐value <0.05 was used as a threshold for significance. The receiver‐operating characteristic (ROC) curve was generated by the R package “pROC”^23^ to evaluate the ability of CYP2E1 to diagnose glioma. Moreover, through The Human Protein Atlas (HPA) database (https://www.proteinatlas.org/), the protein expression of CYP2E1 was analyzed in normal brain tissue and glioma tissues.

### 2.4 RNA extraction and quantitative real‐time PCR

The extraction of CYP2E1 RNA from tissues and cells was carried out using TRIzol reagent (Invitrogen). The PrimeScript RT Reagent Kit (RR047A; Takara) was used to synthesize cDNA. SYBR Premix Ex Taq II (RR820A; Takara) and Bio‐Rad CFX Manager 2.1 real‐time PCR Systems (Bio‐Rad) were used to detect the CYP2E1 mRNA levels following the specifications provided by the manufacturers. The relative Ct method was used to compare the data of the experimental and control groups, and GAPDH was used as the internal control. The primer sequences of mRNA included the following: GAPDH 5′‐GGAGCGAGATCCCTCCAAAAT‐3′ (Forward) and 5′‐GGCTG TTGTCATACTTCTCATGG‐3′ (Reverse); CYP2E1 5′‐ATGTCTGCCCTCGGAGTCA‐3′ (Forward) and 5′‐CGATGATGGGAAGCGGGAAA‐3′ (Reverse).

### 2.5 Correlation of CYP2E1 with clinicopathologic characteristics

The Wilcoxon test investigated the relationship between the expression of CYP2E1 and clinical subtypes in TCGA and CGGA cohorts. (*p*‐value <0.05 was considered as significant). The 587 samples in TCGA and 681 samples in CGGA were divided into two groups according to WHO grade, age (the cutoff was 45 years old), IDH mutation status, 1p19q‐codeletion status, and sex. The level of CYP2E1 in different groups is shown in box plots plotted by the R package “ggpubr” (https://cran.r‐project.org/web/packages/ggpubr/index.html).

### 2.6 Investigation of the prognostic value of CYP2E1 in glioma subtypes

According to the median expression value of CYP2E1, the tumor samples were divided into high and low expression groups in the training and validation sets. Kaplan–Meier (K‐M) survival curves of different subtypes of glioma were generated with the R package “survival” (https://CRAN.R‐project.org/package=survival), and ROC curves were used to evaluate the predictive ability of 1‐, 3‐, and 5‐year overall survival (OS). Furthermore, a K–M curve for disease‐free survival (DFS) in glioma was obtained from the Gene Expression Profiling Interactive Analysis database (GEPIA, http://gepia.cancer‐pku.cn/). Then, to identify independent risk factors for the poor OS of glioma patients, univariate and multifactorial Cox proportional hazards regression analyses were, respectively, performed in the training and validation sets. A *p*‐value <0.05 in univariate Cox regression analysis was selected in multifactorial Cox analysis, and a two‐tailed *p*‐value below 0.05 was considered to be significant.

### 2.7 Protein–protein interaction network (PPI) and functional enrichment analysis

The PPI network of CYP2E1 was predicted by STRING (https://string‐db.org/), and a correlation coefficient of >0.65 and a *p*‐value below 0.01 were considered significantly coexpressed. Then, Gene Ontology (GO) and Kyoto Encyclopedia of Genes and Genomes (KEGG) analyses were performed using the R package “clusterProfiler”^24^ to investigate the potential functions and signaling pathways of coexpressed genes.

### 2.8 Single sample gene set enrichment analysis

In the TCGA data set, 29 immune signatures representing diverse immune cell types, functions, and pathways were quantified to determine their degrees of enrichment in the glioma samples using ssGSEA. Furthermore, the gene sets “WP_Ferroptosis” and “REACTOME_METABOLISM_OF_LIPIDS” were obtained from the Molecular Signatures Database (http://www.broad.mit.edu/gsea/msigdb/). ssGSEA by the R package “GSVA”^25^ was used to evaluate the enrichment scores (ES) of “29 immune signatures,” “ferroptosis,” and “lipid metabolism” for each tumor sample.

### 2.9 Tumor‐infiltrating immune cell profiles

Through the “cibersort”^26^ algorithm, the abundance of TIICs in each glioma sample was evaluated to determine the association between CYP2E1 and TIICs. In addition, correlation analysis among vital immune checkpoints (including PDCD1, CD274, and CTLA4) and CYP2E1 was performed in both TCGA glioma and CGGA mRNA_array_301 cohorts.

### 2.10 Predicting regulatory mirna of CYP2E1

The regulatory miRNAs of CYP2E1 were predicted using two prediction databases: MiDRB (http://www.mirdb.org/) and TargetScan v7.2 (http://www.targetscan.org). Furthermore, correlation analysis between the miRNA predicted and CYP2E1 was performed in TCGA glioma. miRNAs whose expression negatively correlated with the expression of CYP2E1 were defined as potential regulatory miRNAs for this mRNA in gliomas.

### 2.11 DNA amplification and hypomethylation analysis

To further explore the potential mechanism by which CYP2E1 regulates glioma, its genetic and epigenetic changes were explored in glioma samples in TCGA with the cBioPortal database (http://www.cbioportal.org/) according to the tumor samples (*n* = 556) with mRNA data, copy number variation (CNV) data, and DNA methylation data. Linear regression analyses were performed among the mRNA expression level of CYP2E1 and the expression level and methylation level of CNV. A *p*‐value of <0.05 was considered significant.

### 2.12 Analysis of network pharmacology and molecular docking

According to the Traditional Chinese Medicine Systems Pharmacology Database and Analysis Platform (TCMSP, http://tcmspw.com/tcmsp.php), the ingredients in traditional Chinese medicine (TCM) that target CYP2E1 were considered candidates for further analysis through AutoDock 4.2 software, which was used to validate the network pharmacology screening results by docking the active compound with the CYP2E1 protein. The results of molecular docking among effective ingredients and proteins were generated using *PyMOL* software version 2.0.6 (Schrödinger, LLC).

## 3 RESULTS

### 3.1 Downregulation of CYP2E1 expression in gliomas

**FIGURE 1:**
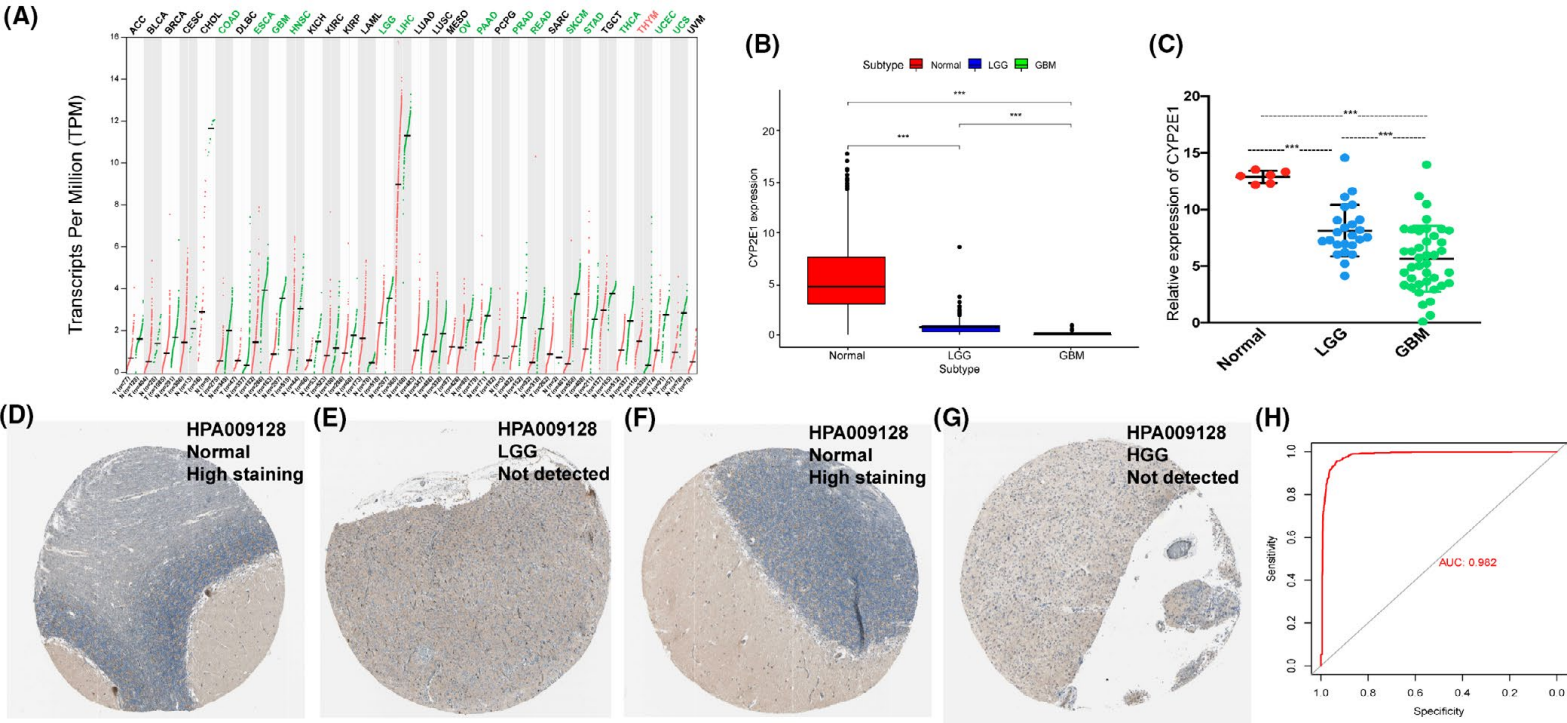
CYP2E1 expression levels in various tumor tissues and the evaluation of its diagnostic value in glioma. (A) CYP2E1 mRNA expression in different normal human tissues and cancer tissues. Green dots represent the expression value in normal tissues, whereas red dots represent the expression value in tumor tissues. (B) Comparison of CYP2E1 mRNA expression in normal tissues and cancer tissues (including LGG and GBM) in the training set. (C) The level of CYP2E1 in LGG and GBM in the validation set. LGG: lower‐grade glioma, GBM: glioblastoma. (D) Representative IHC images of CYP2E1 in (D) normal brain tissue, (E) LGG tissue, (F) normal tissue, and (G) HGG tissue. (H) The mRNA expression of CYP2E1 in the normal brain, LGG, and GBM patients in our hospital. HGG: higher grade glioma. (I). ROC curve analysis revealed that the downregulation of CYP2E1 had high sensitivity and specificity to diagnose glioma (AUC = 0.982) (ns: no significance, *p < 0.05, **p<0.01, ***p < 0.001)

The pan‐cancer analysis showed that the CYP2E1 expression level was lower in most solid cancers than normal tissues but only upregulated in thyroid carcinoma (Figure 1A). The CYP2E1 expression level was significantly lower in glioma tissues in the training set (Figure 1B) and decreased with glioma malignancy. Then, we assessed samples collected in our hospital and found that the level of CYP2E1 was significantly downregulated in glioma tissues compared with normal brain tissues and significantly decreased in GBM compared with lower‐grade glioma (LGG) (Figure 1C). These results indicated that CYP2E1 might be involved in the inhibition of tumor malignancy. Based on IHC staining data of HPA, normal brain tissue had intense CYP2E1 staining, while CYP2E1 was not detected in either lower‐ or higher‐grade gliomas (Figure 1D–G), which was consistent with the trend of mRNA levels. To further explore the diagnostic value of CYP2E1 in gliomas, the area under the receiver‐operating characteristic curve for diagnosing glioma using the level of CYP2E1 was 0.982 in the training set (Figure 1H).

### 3.2 CYP2E1 was correlated with patient clinical characteristics

**FIGURE 2:**
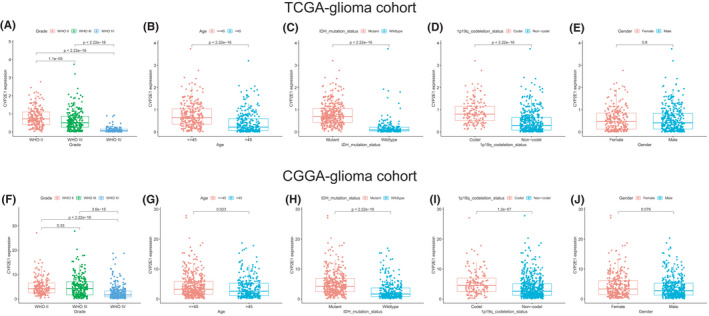
The association between CYP2E1 and clinicopathologic characteristics. In the TCGA cohort, CYP2E1 expression levels were investigated in different (A) WHO grades, (B) age groups, (C) IDH statuses, (D) 1p19q codeletion states, and (E) sex. In the CGGA cohort, the expression levels of CYP2E1 were investigated in different (F) WHO grades, (G) age groups, (H) IDH statuses, (I) 1p19q codeletions, and (J) sex. ***p < 0.001, **p < 0.01, *p < 0.05, NS: not significant

According to the median value, the CYP2E1 mRNA expression level was designated as “low expression” or “high expression.” In TCGA, the level of CYP2E1 decreased with increasing WHO grade (II–IV) of glioma and correlated with the clinical characteristics, including age, 1p19q.‐codeletion status, and IDH mutation status (Figure 2A–D). In the CGGA cohort, the CYP2E1 level was not significantly different among patients with lower WHO grades (WHO II vs. WHO III), and the remaining results were consistent with previous TCGA results (Figure 2F–I). No differences were observed among different genders in either TCGA or CGGA sets (Figure 2E,J).

### 3.3 Downregulation of CYP2E1 expression was correlated with a poor prognosis

**FIGURE 3:**
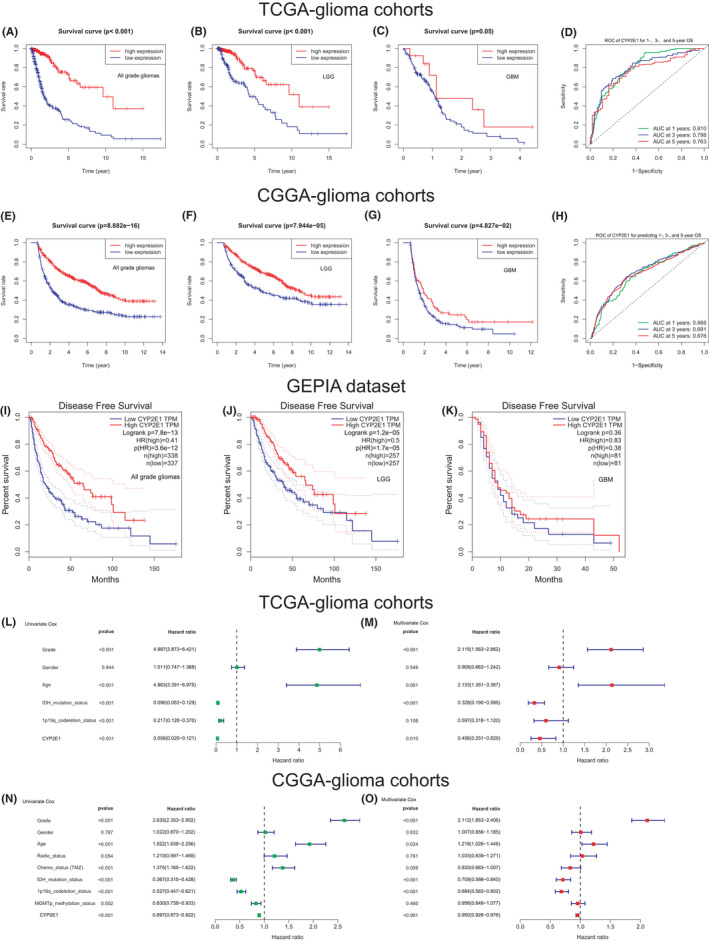
The prognostic value of CYP2E1 in glioma. According to the median value of CYP2E1 expression, patients were divided into low and high expression groups. In the TCGA glioma cohort, K–M curves were generated to investigate the correlation between CYP2E1 expression and OS in (A) all‐grade gliomas, (B) LGG, and (C) GBM. (D) The AUCs of the ROC curve for predicting the 1‐, 3‐, and 5‐year OS of patients in TCGA were 0.810, 0.798, and 0.763, respectively. In the CGGA cohort, K–M curves were generated for (E) all‐grade glioma, (F) LGG, and (G) GBM. (H) The AUCs of the ROC curve for predicting the 1‐, 3‐, and 5‐year OS of patients in CGGA were 0.668, 0.671, and 0.696, respectively. According to the GEPIA database, K–M survival analyses were performed to explore the correlation between CYP2E1 expression and DFS in (I) all‐grade glioma, (J) LGG, and (K) GBM. Forest plot with hazard ratios from the univariate Cox proportional hazards regression analysis in the (L) training set and (M) validation set. Multivariate Cox proportional hazards regression analysis in the (N) TCGA and (K) CGGA cohorts. OS: overall survival, LGG: lower‐grade glioma, GBM: glioblastoma, DFS: disease‐free survival

K–M survival analysis of different glioma subtypes in both TCGA and CGGA cohorts indicated that downregulated expression of CYP2E1 was significantly associated with poor OS and DFS of patients. In the TCGA cohort, as shown in Figure 3A–C, the patients in the low expression group had worse OS than those in the high expression group, including in all WHO grade glioma, lower‐grade glioma (LGG, WHO II, and WHO III), and GBM groups. The AUCs of the ROC curve for predicting 1‐, 3‐, and 5‐year OS according to the value of CYP2E1 were 0.810, 0.798, and 0.763, respectively (Figure 3D). In the CGGA cohort, lower expression of CYP2E1 was significantly associated with poor survival, as shown in Figure 3E–G, and the AUCs of the ROC curve for predicting 1‐, 3‐, and 5‐year OS in CGGA were 0.668, 0.691, and 0.676, respectively (Figure 3H). Figure 3I–K shows that patients in the high expression group had better DFS than those in the low expression group among all grade and LGG groups, while there was no significant difference in DFS among the GBM subtypes according to the GEPIA database. In Figure 3L,M, the univariate Cox logistic regression analysis performed in both TCGA and CGGA identified the value of CYP2E1, grade, and IDH. Status, age, and 1p19q codeletion status were prognostic factors for patients with glioma. Multivariate Cox logistic regression analysis in the two cohorts further confirmed that CYP2E1 expression could be an independent factor in predicting glioma patients’ prognosis (Figure 3N,O). These results demonstrated that the downregulation of CYP2E1 indicated a poorer prognosis for patients.

### 3.4 PPI network and functional analysis

**FIGURE 4:**
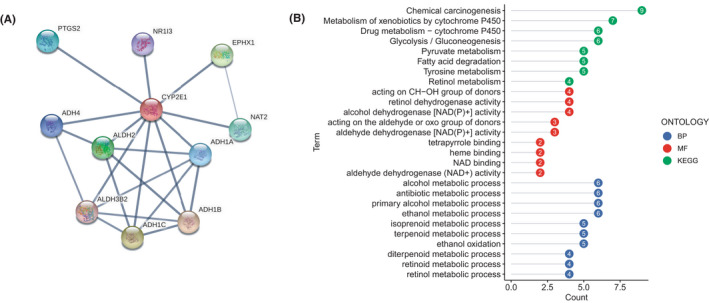
Coexpression network and potential function of CYP2E1. (A) Protein–protein interaction (PPI) networks. (B) GO and KEGG analyses and significantly enriched terms of coexpressed genes in glioma

As shown in Figure 4A, ADH1A, ADH1B, ADH1C, ADH4, ALDH2, ALDH3B2, EPHX1, NAT2, PTGS2, and NR1I3 were considered to be coexpressed with CYP2E1. The proteins encoded by most of the genes were members of the alcohol dehydrogenase (ADH) family. PTGS2, known as cyclooxygenase 2, is a typical ferroptosis indicator.^27^ GO and KEGG analyses indicated that these coexpressed genes were mainly involved in ethanol metabolic processes and lipid metabolism‐related pathways (Figure 4B). These results suggested that CYP2E1 may be involved in the regulatory mechanism of lipid metabolism and ferroptosis‐related biologic processes.

### 3.5 Results of SSGSEA

**FIGURE 5:**
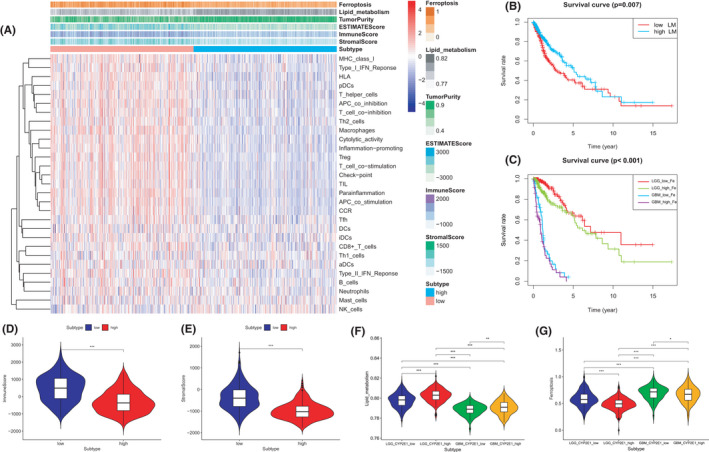
Single sample gene set enrichment analysis (ssGSEA) in TCGA glioma samples. Patients were assigned to low and high expression groups according to the median value of CYP2E1. (A) Heatmap represents the enrichment scores (ES) from ssGSEA of ferroptosis, lipid metabolism, and immune‐related hallmarks in each tumor sample. K–M survival analysis for the ES of lipid metabolism (B) and ferroptosis (C) pathways in glioma samples. (D) Differential immune score (D) and stromal score (E) analysis among CYP2E1 low and high expression groups. By cutoff line (median value of CYP2E1) and subtype of gliomas (LGG and GBM). Samples were assigned into four groups: LGG low CYP2E1 expression, LGG high CYP2E1 expression, GBM low CYP2E1 expression, and GBM high CYP2E1 expression groups. ES of lipid metabolism (F) and ferroptosis (G) differences among the four groups. LM: lipid metabolism, Fe: ferroptosis. ***p < 0.001, **p < 0.01, *p < 0.05, NS: not significant

Glioma samples were assigned to low and high expression groups according to the median value of CYP2E1 expression. The ssGSEA score was used to quantify the activities or abundances of the immune signatures, lipid metabolism, and ferroptosis in TCGA glioma samples. In Figure 5A, the ES of immune signatures in the low expression group were significantly higher than those in the high expression group, and the infiltrating level of TIICs, including the stromal and immune scores, was also higher in the low CYP2E1 expression group. Moreover, samples with lower tumor purity were more common in the low expression group. In addition, to explore the influence of lipid metabolism and ferroptosis on prognosis in glioma, survival analysis was performed and indicated that a higher ES of lipid metabolism was correlated with a better OS for patients (Figure 5B). In contrast, for the “ferroptosis” term, higher ES was associated with a more inferior OS in both LGG and GBM (Figure 5C). The box plot in Figure 5D,E confirmed that higher immune scores and stromal scores were negatively correlated with the level of CYP2E1. As Figure 5F shows, samples in LGG had a higher ES of lipid metabolism than those in GBM, whereas in both the LGG and GBM subtypes, a higher ES was positively associated with a higher expression level of CYP2E1. In Figure 5G, the difference analysis of ferroptosis ES among different groups indicated that patients with GBM had higher ES than patients with LGG. In addition, among GBM and LGG, samples with higher ES of ferroptosis were more common in the lower CYP2E1 expression level groups. These results indicated that CYP2E1 might be involved in the signaling pathways of lipid metabolism and ferroptosis. Furthermore, in the LGG or GBM subtype, downregulation of CYP2E1 was positively correlated with relatively active ferroptosis and an inactive lipid metabolism‐related pathway.

### 3.6 TIIC profiles

**FIGURE 6:**
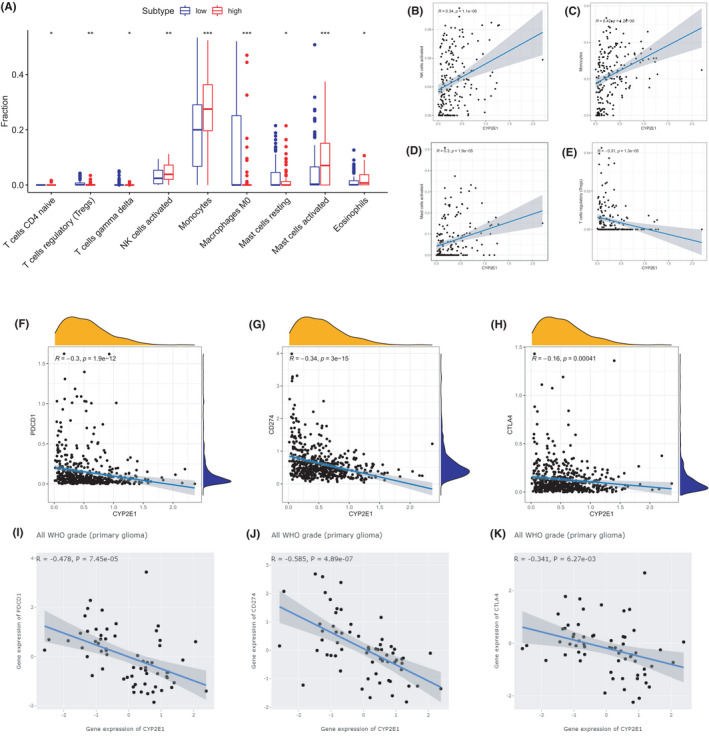
The correlation between immunity and CYP2E1 in TCGA glioma. (A) The abundance of tumor‐infiltrating immune cells (TIICs) in the high and low expression groups of CYP2E1 in glioma in TCGA (immune cells with *p*‐value <0.05 were shown). Correlation analysis between CYP2E1 expression and (B) activated NK cells, (C) monocytes, (D) activated mast cells, and (E) regulatory T cells (Tregs). The expression of CYP2E1 was negatively correlated with the expression of (F, I) PDCD1, (G, J) CD274, and (H, K) CTLA4 according to the Spearman correlation analysis of TCGA and CGGA cohorts. ***p < 0.001, **p < 0.01, *p < 0.05, NS: not significant

The relationship between CYP2E1 and the TIME in terms of the abundance of immune cells was investigated. Here, we just showed the abundance of immune cells among low and high expression groups with *p*‐value <0.05 in the difference analysis. As shown in Figure 6A, the high CYP2E1 expression group had a higher fraction of activated NK cells, monocytes, activated mast cells, and eosinophils than the low CYP2E1 expression group. Correlation analysis (Figure 6B–E) showed that the level of CYP2E1 was positively correlated with activated NK cells (rho = 0.34, *p* < 0.001), monocytes (rho = 0.42, *p *< 0.001), and activated mast cells (rho = 0.3, *p*< 0.001) and negatively associated with regulatory T cells (Tregs, rho = −0.31, *p *< 0.001). Moreover, vital immune checkpoint (PDCD1, CD274, and CTLA4) expression levels were significantly negatively correlated with the level of CYP2E1 mRNA in both TCGA and CGGA cohorts. The rho and p‐values of each result are shown in Figure 6F–K. These results indicated that the proportion of immune cells and immune checkpoints was associated with the level of CYP2E1 mRNA expression.

### 3.7 Targeting miRNA, DNA amplification, and DNA hypomethylation contributes to CYP2E1 downregulation in glioma

**FIGURE 7:**
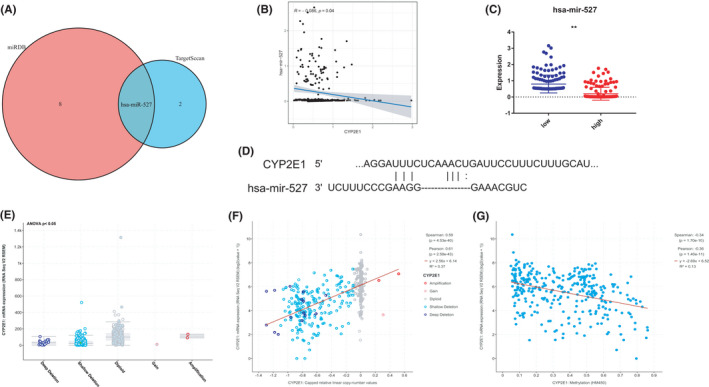
Regulatory network analysis of CYP2E1. (A) Selection of potential regulatory miRNAs of CYP2E1 in the predicted cohorts. (B) The correlation between hsa‐miR‐527 expression and CYP2E1 mRNA expression in the TCGA glioma cohort. (C) The difference analysis of has‐miR‐527’ expression in CYP2E1‐low and high expression group. (D) The putative binding site of CYP2E1 3′UTR by hsa‐miR‐527. (E) Box plots of CYP2E1 mRNA expression in glioma tissues indicating genetic status. Correlation analysis between CYP2E1 mRNA expression and CYP2E1 CNV numbers (F) and DNA methylation (G)

To further explore the mechanism by which CYP2E1 is dysregulated in gliomas, we analyzed its targeting miRNA and genetic and epigenetic alterations in TCGA glioma. According to the miRDB and TargetScan databases, hsa‐miR‐527 was considered the regulatory miRNA for CYP2E1 (Figure 7A). Further, we found a negative association between expression levels of CYP2E1 and has‐miR‐527 in the TCGA cohort (Figure 7B,C). The correlation confirmed the regulatory relationship of their expressions, the upregulation of has‐miR‐527 would lead to the downregulation of CYP2E1 mRNA in gliomas. Figure 7D showed the miRNA binding site within the CYP2E1 mRNA target. In addition, in tumor samples with mRNA data and CNV data, shallow deletion was significantly associated with downregulation of CYP2E1 mRNA (ANOVA *p *< 0.05) (Figure 7E). Moreover, the value of CYP2E1 increased with the capped relative linear copy number values of CYP2E1 (Figure 7F, Pearson's *r* = 0.61, *p *< 0.0001). Furthermore, linear regression analyses indicated that the total methylation level of CYP2E1 was moderately and negatively correlated with mRNA expression (Figure 7G, Spearman's *r* = −0.34, Pearson's *r* = −0.36, *p *< 0.0001).

### 3.8 Components of TCM targeting CYP2E1

**FIGURE 8:**
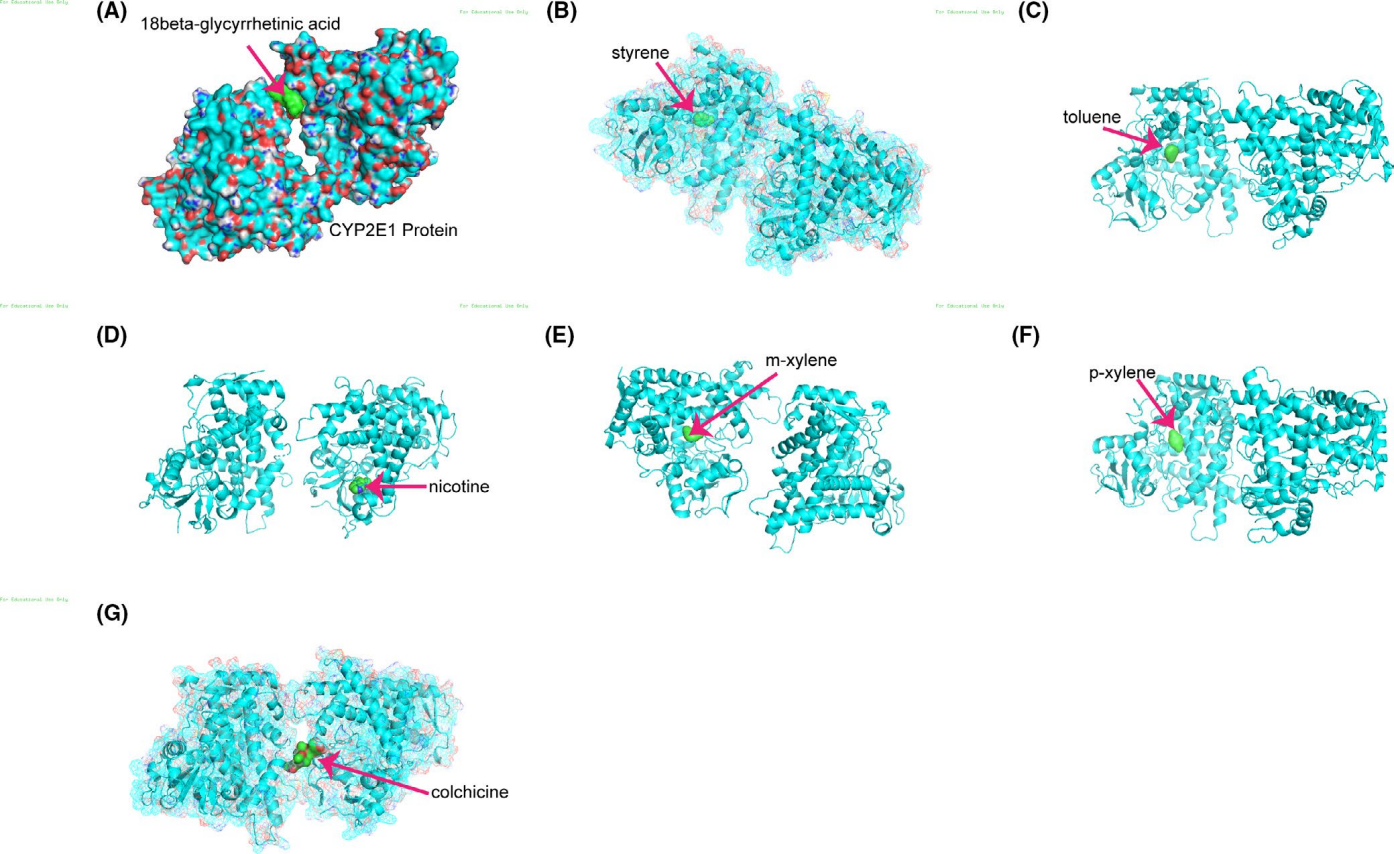
Ingredients in related traditional Chinese medicines that target the CYP2E1 protein. The molecular docking of CYP2E1 and 18beta‐glycyrrhetinic acid (A), styrene (B), toluene (C), nicotine (D), m‐xylene (E), p‐xylene (F), and colchicine (G)

Effective compounds targeting CYP2E1, including 18beta‐glycyrrhetinic acid, styrene, toluene, nicotine, m‐xylene, p‐xylene, and colchicine, were identified. A molecular docking study was performed to determine the putative docking of CYP2E1 with the above effective compounds, as shown in Figure 8A–G.

## 4 DISCUSSION

The identification of novel glioma biomarkers is vital for studying molecular mechanisms and improving patient prognosis. CYP2E1, identified as critical alcohol metabolizing enzyme, has not yet been systematically studied in gliomas. Previous studies reported that CYP2E1, which is involved in the bioactivation of urethane, decreased urethane metabolism in vivo and was relatively resistant to urethane‐induced lung cancers.^28^, ^29^ In our study, CYP2E1 was identified as a tumor suppressor gene associated with glioma diagnosis and prognosis and could distinguish clinical molecular characteristics among glioma samples. CYP2E1 could be considered a novel marker of glioma, which is an important discovery related to its great significance in tumors. Moreover, the findings were verified in multiple databases and at types of omics (mRNA and protein).

Recently, alterations in lipid metabolism have emerged as a typical phenotype of most carcinomas.^30^ In addition, ferroptosis is a lipid peroxidation‐dependent and iron‐dependent form of cell death.^31^ Moreover, some mechanisms related to immunity are tightly linked to cellular lipid metabolism.^32^ An analysis of the effect of lipid metabolism on TIIC functions was conducted to examine a potential association between immune regulation and cellular lipid metabolism.^33^ In this study, we investigated the effect of CYP2E1 on lipid metabolism and TIME to explain the underlying mechanism of the inhibition of glioma malignancy. First, CYP2E1 expression was found to correlate with the regulation of lipid metabolism in glioma. As the level of CYP2E1 mRNA decreased, the activity of the lipid metabolic pathway showed a downward trend, while a lower lipid metabolism ES showed a worse prognosis. This finding is consistent with previous reports, and lipid metabolism is a vital molecular mechanism related to prognosis.^34^ Downregulated CYP2E1 expression, which is associated with lipid metabolism, also promotes glioma progression. Furthermore, considering that ferroptosis is regulated by the production of lipid oxidation, we explored the association between these processes and found that the downregulation of CYP2E1 expression was correlated with the active ferroptosis signaling pathway. These results indicated that CYP2E1 might affect the malignant behavior, proliferation, and progression of glioma by regulating ferroptosis and lipid metabolism pathways.

Subsequently, based on the characteristics of the immune microenvironment, the effects of CYP2E1 on glioma invasion and growth were explored in this research. We found a significant positive correlation between CYP2E1 expression and tumor‐killing immune cells. NK cells cooperate with T cells to restrain tumor growth,^35^ monocytes play an important antitumor role as antigen‐presenting cells,^36^ and some researchers have reported that infiltration of mast cells in the tumor is associated with better patient survival.^37^ In addition, although CYP2E1 was highly correlated with monocyte infiltration, there was no significant correlation between the induction of M1 or M2 tumor‐associated macrophages from monocytes under different conditions. This result suggested that CYP2E1 may not be involved in regulating monocyte differentiation to exert its effects further. However, to escape being distinguished and killed by the immune system, cancers may use various methods to suppress the function of infiltrating immune cells.^38^ Downregulation of CYP2E1 expression was positively correlated with the abundance of Tregs. As the main immunosuppressive TIICs, Tregs can promote the escape and progression of cancers by inhibiting immune cell aggregation and antitumor effects. In addition, the negative correlation between CYP2E1 and immune checkpoints also proved that downregulation of CYP2E1 expression might be related to the immunosuppressive characteristics of the microenvironment in glioma.^39^ Tumors can exploit the connection between immune cell metabolism and function to suppress immunity and promote their progression,^38^ as represented in other reports. As a metabolism‐related gene, the expression of CYP2E1 is also correlated with the immune microenvironment.

The underlying mechanisms of CYP2E1 dysregulation in cancers have not been fully elucidated. Genetic aberrations of tumor suppressor genes have been considered a breakpoint in tumorigenesis.^40^ Consistent with this, we further examined the association between CYP2E1 DNA methylation and CYP2E1 mRNA expression. The results indicated that hypermethylation was significantly associated with the downregulation of CYP2E1 expression. Moreover, miRNAs are critical regulators of gene expression that can downregulate target genes by inducing mRNA degradation or translation obstruction by binding the 3′‐UTR of the target mRNA.^41^ In this research, we found that hsa‐miR‐527 expression was significantly negatively correlated with CYP2E1 mRNA expression. These findings indicate that genetic and epigenetic alterations (including methylation and alteration of CNV) contribute to CYP2E1 dysregulation in gliomas.

In addition, we identified seven TCM drugs that target CYP2E1. Recent research has provided evidence that natural active ingredients in TCM drugs have practical antitumor therapeutic effects on solid tumors.^42^ Network pharmacology has been extensively applied by researchers.^43^ 18beta‐glycyrrhetinic acid, styrene, toluene, nicotine, m‐xylene, p‐xylene, and colchicine may play a role in gliomas by influencing CYP2E1, which requires further study.

Our study found that CYP2E1 expression was significantly downregulated in gliomas and might be a potential prognostic biomarker related to the OS and DFS of patients. In addition, the activity of lipid metabolism and the ferroptosis pathway may be related to the expression level of CYP2E1. However, the specific mechanism needs to be further verified. In addition, CYP2E1 is related to the immunosuppressive microenvironment, which explains the correlation between its metabolism‐related function and immunity. This research also shows that CYP2E1 could affect the progression and invasion of glioma cells through a variety of possible mechanisms, which confirms the great significance of research about this molecule. In addition, we tried to explore the potential regulatory mechanism of CYP2E1 from the perspectives of epigenetic and DNA modification disorders. Glioma cells may downregulate the expression of CYP2E1 through methylation modification and DNA copy variation. The upstream miRNA might also specifically target CYP2E1 to regulate its expression at mRNA level. No research has been conducted to investigate the carcinogenesis of CYP2E1 via ferroptosis regulation pathways in gliomas. Hence, it would be of great significance to further elucidate the underlying mechanisms in future.

## 5 CONCLUSION

In general, CYP2E1 expression was significantly downregulated in glioma tissues relative to normal brain tissues. Overexpressed CYP2E1 could independently predict better OS and RFS in patients with glioma. Furthermore, we proved that CYP2E1 is related to lipid metabolism, ferroptosis, and the immune microenvironment. DNA amplification, methylation, and hsa‐miR‐527 could be the mechanisms associated with CYP2E1 dysregulation in gliomas. In addition, seven practical components of Chinese medicine were predicted to target CYP2E1. This study identified a novel biomarker of glioma and provided a new perspective for understanding the mechanism underlying its function in gliomas.

## ETHICS STATEMENT

Institutional Ethics Committee of the Faculty of Medicine at Renmin Hospital of Wuhan University approval (2012LKSZ (010) H) to carry out the study within its facilities. Ethical approval was waived since we used only publicly available data in this study.

## CONFLICT OF INTEREST

The authors declare that they have no conflicts of interest.

## Data Availability

Publicly available data sets were analyzed in this study. This data can be found below:
TCGA, https://www.cancer.gov/, CGGA, http://www.cgga.org.cn/, and STRING, https://string‐db.org/cgi/input.pl
